# Can we rely on selected genetic markers for population identification? Evidence from coastal Atlantic cod

**DOI:** 10.1002/ece3.4648

**Published:** 2018-12-01

**Authors:** Per Erik Jorde, Ann‐Elin Synnes, Sigurd Heiberg Espeland, Marte Sodeland, Halvor Knutsen

**Affiliations:** ^1^ Department of Biosciences, Centre for Ecological and Evolutionary Synthesis University of Oslo Oslo Norway; ^2^ Institute of Marine Research His Norway; ^3^ Centre of Coastal Research University of Agder Kristiansand Norway

**Keywords:** marine fishes, natural selection, population genetics, population of origin, statistical assignment

## Abstract

The use of genetic markers under putative selection in population studies carries the potential for erroneous identification of populations and misassignment of individuals to population of origin. Selected markers are nevertheless attractive, especially in marine organisms that are characterized by weak population structure at neutral loci. Highly fecund species may tolerate the cost of strong selective mortality during early life stages, potentially leading to a shift in offspring genotypes away from the parental proportions. In Atlantic cod, recent genetic studies have uncovered different genotype clusters apparently representing phenotypically cryptic populations that coexist in coastal waters. Here, we tested if a high‐graded SNP panel specifically designed to classify individual cod to population of origin may be unreliable because of natural selection acting on the SNPs or their linked background. Temporal samples of cod were collected from two fjords, starting at the earliest life stage (pelagic eggs) and carried on until late autumn (bottom‐settled juveniles), covering the period during summer of high natural mortality. Despite the potential for selective mortality during the study period, we found no evidence for selection, as both cod types occurred throughout the season, already in the earliest egg samples, and there was no evidence for a shift during the season in the proportions of one or the other type. We conclude that high‐graded marker panels under putative natural selection represent a valid and useful tool for identifying biological population structure in this highly fecund species and presumably in others.

## 1 INTRODUCTION

**Figure 1:**
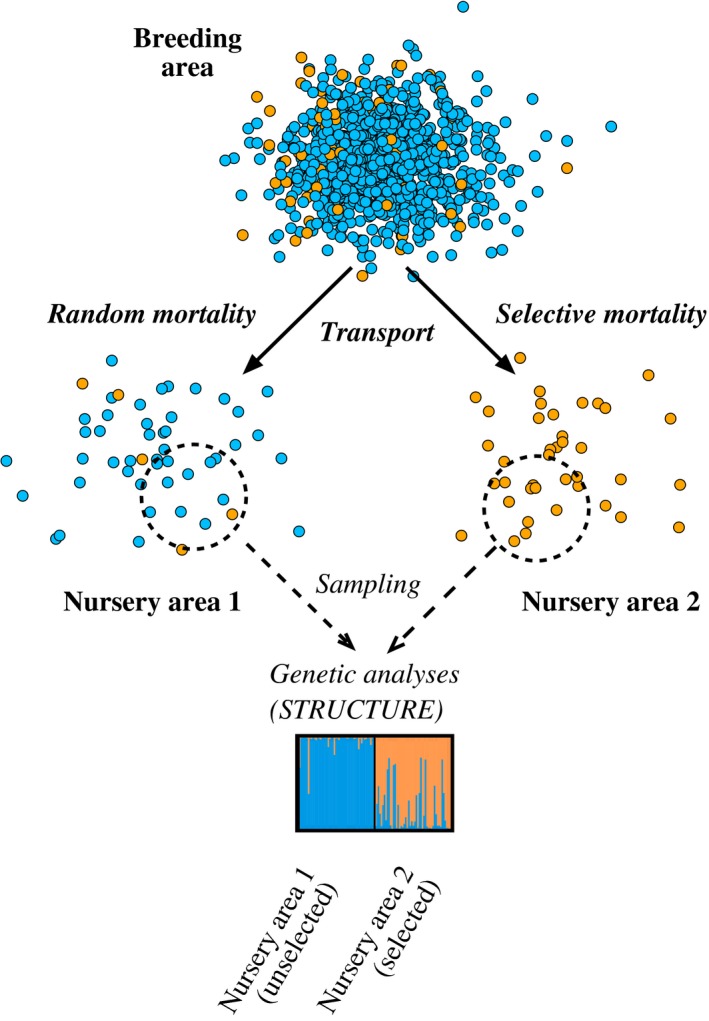
Hypothetical scenario of a breeding population distributing juveniles (e.g., seeds or larvae) to two nursery areas that differ in environmental conditions and thus in selective mortality. Selection is assumed to favor individuals that are homozygote in three particular loci (identified as orange dots) and in one nursery area (area 2) but not in the other (area 1). Below is a *Structure* plot of samples from the two hypothetical nursery areas. See Supporting Information for details

In order to increase statistical power to resolve weak population genetic structure, a select panel of loci with higher than average level of genetic differentiation is often employed (André et al., 2011; Banks, Eichert, & Olsen, 2003; Henriques et al., 2018; Johansen et al., 2018; Jorde, Kleiven, et al., 2018; Larson, Seeb, Pascal, Templin, & Seeb, 2014; Nielsen et al., 2012; Russello, Kirk, Frazer, & Askey, 2012). Such a high‐graded panel is likely to include loci under divergent selection, raising concerns over their reliability as a tool for inferring demographic population structure (Luikart, England, Tallmon, Jordan, & Taberlet, 2003; Nielsen, Hansen, & Meldrop, 2006). Selected loci may nevertheless be excellent tools for the more restricted purpose of discriminating populations (Bekkevold et al., 2015; Lamichhaneya et al., 2012; Milano et al., 2014; Teacher, André, Jonsson, & Merilä, 2013) and for assigning individuals to population of origin (Banks et al., 2003; Freamo, O'Reilly, Berg, Lien, & Boulding, 2011; Helyar et al., 2011; Kavakiotis, Samaras, Triantafyllidis, & Vlahavas, 2017; Nielsen et al., 2012; Wilkinson et al., 2011). Challenges arise when selection on the markers is strong enough for environmental differences to override population demography on allele frequency dynamics. Individuals and genotypes sampled after an episode of selective mortality may poorly represent the parental generation and could lead to false impressions of population structuring. Such a scenario is illustrated in Figure 1, depicting the outcome of hypothetical selective mortality on genotype composition following transport of juveniles to different nursery areas. Upon sampling and genetic screenings of samples from the nursery areas, the results indicate genetically distinct groups that may be mistaken for separate biological populations, which they are not. While strong selection acting on a single or small number of marker loci is unlikely to have a great overall effect on a large panel of markers, the situation is different when using a small set specifically chosen for their high levels of divergence. This could be a problem especially when the true population structure is weak, absent or even moderate, as selection may generate patterns of genetic structure that trace environmental drivers rather than population processes (Lamichhaneya et al., 2012; Nielsen et al., 2006).

Strong selection in the form of non‐random survival of genotypes is not unreasonable in organisms that combine extremely high fecundity with widespread dispersal of offspring into a diverse range of environments. High fecundity implies a high reproductive excess, for some organisms in the millions (Winemiller & Rose, 1992). To maintain population size, this excess must be balanced by high mortality, usually at early life stages. Thus, there is a potential for selective mortality in the offspring and the tiny fraction of individuals that survive and end up being sampled for genetic analyses may then poorly represent the parent population. While most mortality is likely to be unrelated to the individual's genotype and thus non‐selective, even when, say, 99.9% of deaths are unrelated to genotype, there remains a reproductive excess on the order of 1,000 to cover the cost of natural selection if the excess was a million to begin with. Many highly fecund species also have a highly dispersive early life stage (e.g., seed plants [Nathan & Muller‐Landau, 2000], marine invertebrates [Grantham, Eckert, & Shanks, 2003], and fishes [Cowen & Sponaugle, 2009]), and offspring may end up in environments their parents were not adapted to. Temporal fluctuations in environmental conditions could also contribute to create a mismatch between parental adaptation and optimal offspring genotypes, creating an option for selective mortality in offspring.

The use of high‐graded markers is particularly attractive for marine organisms because population structure is typically weak within oceans (Hauser & Carvalho, 2008; Waples, 1998; Ward, Woodwark, & Skibinski, 1994). However, many marine species represent precisely the pattern of high fecundity and widespread dispersal followed by massive juvenile mortality that could cause problems for some genetic markers to provide reliable information on biological population structure and for correctly assigning individuals to population of origin. Here, we explore these issues empirically, using a panel of 27 SNP markers that were specifically developed for assigning Atlantic cod (*Gadus morhua*) along the south coast of Norway to population of origin, that is, to putative “North Sea” or “fjord” populations (Jorde, Kleiven, et al., 2018; Knutsen et al., 2018). We tested the hypothesis that such assignments were driven by selective mortality during the early life stages by monitoring genotype composition in eggs and juveniles over the time period (early spring to autumn) with highest natural mortality. The potential for selection on polymorphic loci in this highly fecund species lies in the extensive drift of pelagic eggs and larvae with ocean current and in the potentially contrasting environments where they settle and grow up. The alternative hypothesis is that genetic clustering and assignments of coastal cod is not unduly affected by ongoing selection on the SNP markers.

## 2 MATERIAL AND METHODS

### 2.1 The study species and experimental setting

**Figure 2:**
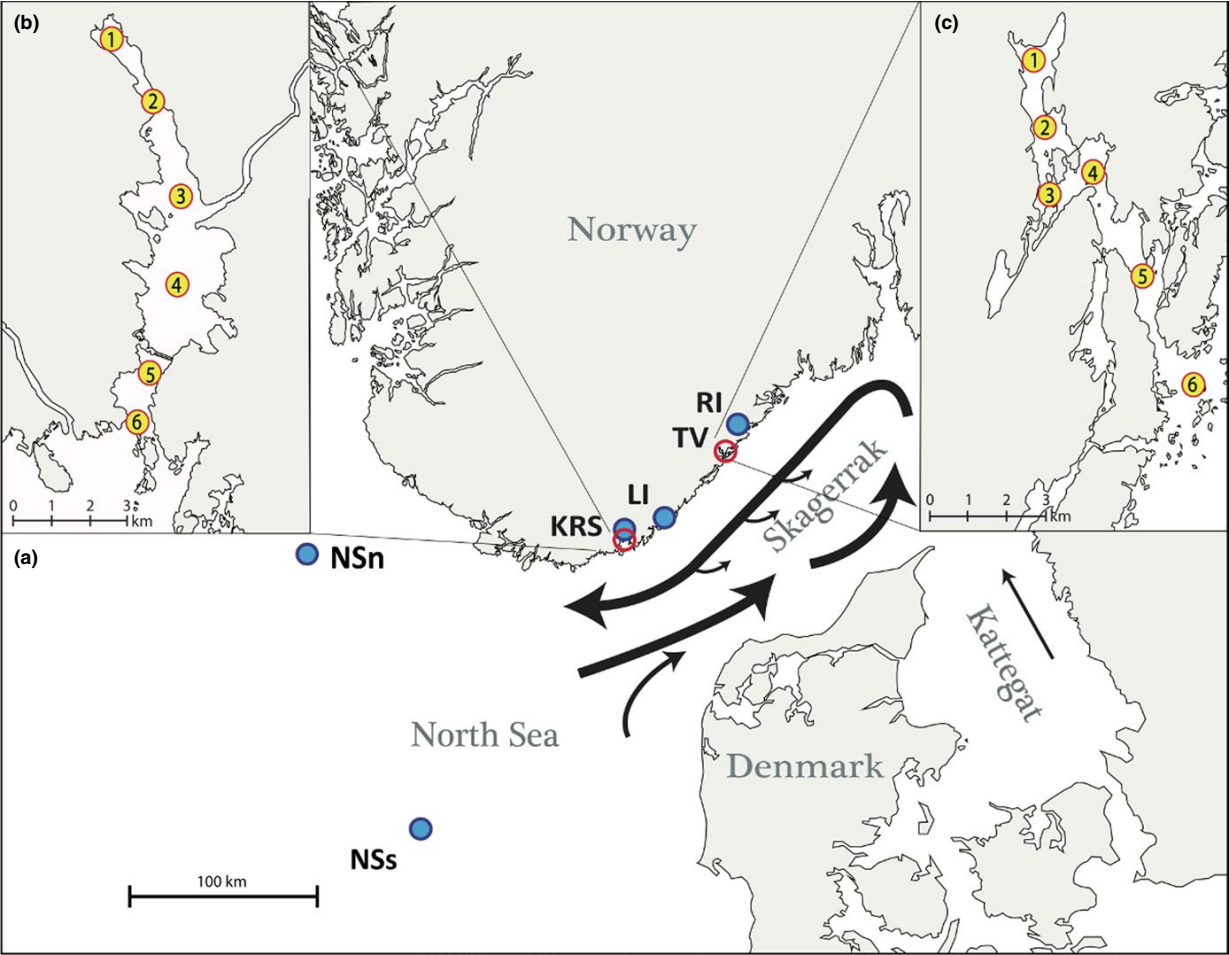
(a) Map of study area with sample locations for eggs and juveniles. Blue dots indicate position of reference samples in the North Sea (NSn and NSs) and within three fjords (KRS: Kristiansand; LI: Lillesand; RI: Risør). Black arrows indicate the dominant ocean currents (simplified from Danielssen et al., 1997). Insets: details of sampled fjords with sample locations (numbered yellow dots): (b) Topdalsfjord; (c) Tvedestrandsfjord

The Skagerrak is an extension of the North Sea, situated between Denmark, Sweden, and southern Norway, bordering Kattegat (Figure 2). Spawning of Atlantic cod occurs in the North Sea, in the Kattegat, and in Skagerrak coastal waters during early spring (February to early April). The Atlantic cod is a highly fecund species, the female producing approximately half a million eggs per kg body weight (Kjesbu, 1989; May, 1967; Oosthuizen & Daan, 1974). Spawning products (eggs and larvae) are pelagic and subject to transport with ocean currents (Munk, Larsson, Danielsen, & Moksness, 1995), which in the Skagerrak form a counter‐clockwise path from the North Sea along the Skagerrak coast (Figure 2). Thus, spawning products from the North Sea can and do reach the Skagerrak coast (Knutsen et al., 2004; Spies et al., 2018; Stenseth et al., 2006), and cod from the outer coastal areas in the Skagerrak appears to be genetically similar to or identical with North Sea cod (André et al., 2016; Barth et al., 2017; Jorde, Kleiven, et al., 2018; Knutsen et al., 2011; 2018; Sodeland et al, 2016). Eggs hatch after three to four weeks (von Westernhagen, 1970) and the larvae remain pelagic until early summer when they descend to the bottom and are referred to as 0‐group. Mortality rates during early life stages of cod have been estimated to approximately 10.9% per day at the early larval stage, declining to 2.2% per day for larger larvae, and considerably lower than this for post‐settled 0‐group cod (Sundby, Bjørke, Soldal, & Olsen, 1989).

Genetic studies of 0‐group and older cod along the Norwegian Skagerrak coast have found genetic differences mainly between inner fjords and outer skerries (Knutsen et al., 2011; Øresland & André, 2008). This spatial pattern of genetic variability has been attributed to the existence in the Skagerrak of genetically distinct forms or putative ecotypes of cod (Barth et al., 2017), co‐occurring in coastal waters (Knutsen et al., 2018; Sodeland et al., 2016). Based on a panel of >9,000 SNPs, Jorde, Kleiven, et al. (2018) developed a small panel of 27 SNPs for cost‐efficient assignment of coastal cod from Skagerrak into two ecotypes, referred to as “fjord cod” and “North Sea cod”, respectively. The panel was developed by ranking loci according to levels of genetic divergence (Nei's *G*_ST_) in their study area, which broadly overlapped the present one, while avoiding closely linked (composite linkage disequilibrium, CLD > 0.5) loci. Thus, the 27 SNP panel represents a high‐graded subset of genetic markers specifically developed to provide high levels of divergence among cod in the present study area.

### 2.2 Study areas

The present study areas include two nearby fjords on the Norwegian Skagerrak coast, the Topdalsfjord and Tvedestrandsfjord (Figure 2). Topdalsfjord (Figure 2b) is located near the city of Kristiansand, and is approximately 11 km long until it opens significantly to the semi‐open sea, and has a largest depth of about 100 m. The fjord is known to hold several eelgrass beds which are considered to be one of the most important nursery areas for Atlantic cod. Tvedestrandsfjord (Figure 2c) is located outside the city of Tvedestrand and is approximately 8 km long with a maximum depth of 85 m. Studies of current patterns in this fjord indicate that pelagic eggs and larvae on average tend to experience an inward transport by estuarine circulations and thus become retained within the inner fjord basins (Ciannelli et al., 2010; Knutsen et al., 2007). Tvedestrandsfjord has recently been protected as a marine protected area (MPA), including a no‐take zone, and fishing mortality during the present study is expected to be negligible.

### 2.3 Sampling

Cod eggs were sampled during the spawning season from February to late March 2015, once in Topdalsfjord and five times in Tvedestrandsfjord. Six sampling sites or “stations” were arranged in the form of transects from the innermost to the outer part of the fjords (Figure 2b,c). Eggs were sampled with a WP2 planktonic net (Fraser, 1968) with 60 cm diameter and 500 µm mesh size. The net was hauled vertically from 30 m depth to the surface at a speed of 0.5 m/s. Eggs were identified and determined to species according to size and pigmentation (Hiemstra, 1962). Cod eggs were considered to be 1.2 mm to 1.5 mm in diameter (Thompson & Riley, 1981). Eggs were stored in 96% ethanol at −22°C until DNA extraction.

Sampling of young‐of‐the‐year juveniles (0‐group) was done first in early summer (June), then once again later in autumn (September and October) in both fjords, using a standardized protocol for the annual beach survey by the Institute of Marine Research (IMR) along the Norwegian Skagerrak coast (Barceló, Ciannelli, Olsen, Johannessen, & Knutsen, 2016). The Topdalsfjord was sampled for juveniles at six different stations, once in June and once in September but the latter employed somewhat different sampling stations (corresponding approximately to stations 3 and 6: Figure 2b) to comply with the annual IMR beach seine program. Tvedestrandsfjord was sampled for juveniles at five stations (no. 1 through 5) in June and three stations (1 through 3: Figure 2c) in October. Juveniles were stored frozen at −22°C until DNA extraction.

Mature, supposedly spawning, cod were sampled from Topdalsfjord during February 2015 with the help from a local fisher. Sampling was done at five different locations within the inner parts of the fjord (approximately stations 1 through 4: Figure 2b) over three days of fishing. Sampled cod were sacrificed, measured, and sexed by visual examination of gonads. A piece of the dorsal fin was saved for genetic analysis and was stored in 96% ethanol at −22°C until DNA extraction.

### 2.4 Reference samples

As genetic references for cod in the study area we used two previously sampled and genotyped sets of individuals from the Norwegian Skagerrak coast and from the North Sea, respectively (Jorde, Kleiven, et al., 2018). The two reference samples consisted of a (*n* = 143) sample of juvenile cod from the inner part of three fjords (including Topdalsfjord and two other nearby fjords, sampled in 1997–2010) and a sample (*n* = 91) of adult cod from two locations (sampled in 2002 and 2012, respectively) in the North Sea (Figure 2).

### 2.5 DNA extraction

Sampled cod eggs were extracted for DNA using the E.Z.N.A MicroElute Genomic DNA Kit (Omega Bio‐tek, Norcross, GA), following the manufacturer's instructions for tissue samples with only one minor modification: the last elution buffer step being done twice through the same filter (25 µl was eluted). Genomic DNA from juvenile and spawning cod was extracted from a small piece of the dorsal fin, using E.Z.N.A Tissue DNA kit (Omega Biotek) following the protocol. DNA from all individual cod samples was quality‐verified and quantified with a NanoDrop instrument (NanoVue Plus, GE healthcare).

### 2.6 Genotyping

A total of 333 cod eggs, 100 young‐of‐the‐year juvenile cod, and 52 adult cod were genotyped for the present study (Table 1). Genotyping of the 27 SNPs was carried out on a Sequenom MassARRAY platform at the Centre for Integrative Genetics, Norway (https://cigene.no). We dismissed individuals with 10 or more missing genotypes as having poor DNA quality, resulting in 76 individuals (70 eggs, 6 juveniles, 0 spawners) being removed from further analyses, which were based on the remaining 409 individuals (Table 1). We consistently got genotypes only from 25 of the 27 SNPs, with two SNPs (ss1712301578 and ss1712299621: www.ncbi.nlm.nih.gov/SNP/) often failing, and all statistical analyses were therefore limited to 25 SNPs.

**Table 1:** The target samples from the Topdalsfjord and Tvedestrandsfjord

Date (DD.MM.YYYY)	Life stage	Sample sizes		*F*_IS_	Assigned to	
		n_1_	n_2_		NS	fjord
Topdalsfjord						
19–25.02.2015	Adult	52	52	0.019	5	47
05.03.2015	Egg	126	120	0.046*	9	111
15.06.2015	Juvenile	10	9	0.080	2	7
15.09.2015	Juvenile	11	10	−0.094	1	9
			*χ* ^2 ^= 2.308, *df *= 3, *p* = 0.511			
Tvedestrandsfjord						
20.02.2015	Egg	7	2	NA	0	2
27.02.2015	Egg	77	46	0.012	0	46
06.03.2015	Egg	61	45	0.094*	11	34
13.03.2015	Egg	33	25	−0.024	3	22
24.03.2015	Egg	29	25	−0.012	1	24
08.06.2015	Juvenile	54	50	0.094	31	19
12.10.2015	Juvenile	25	25	0.038	2	23
			*χ* ^2^ = 69.31, *df *= 6, *p* = 0.000			
Total		485	409		65	344

Note

For each sample are given date of sampling, life stage sampled, sample sizes (*n*_1_ = total number of genotyped individuals; *n*_2_ = number of those that were successfully genotyped, i.e., with <10 genotypes missing), average *F*_IS_ over 25 loci (NA = not calculated due to low sample size; asterisks indicate significance at the 5% level with *Genepop* probability test), and numbers assigned by *Geneclass2* to the “North Sea” (NS) and “fjord” types. *χ* ^2^ refers to the contingency chi‐square test for homogeneity of proportions assigned to the two types at different sample times and life stages.

### 2.7 Statistical analyses

Correlations of alleles within individuals relative to the sample (*F*_IS_) and among samples relative to the total (*F*_ST_) were calculated according to Weir and Cockerham (1984), separately for each SNP and as averages over loci, using the Genepop software (v. 4.2.1: Rousset, 2008). Genotype proportions within samples were tested for conformation to Hardy–Weinberg expectations with the chi‐square goodness‐of‐fit test. Individuals were clustered on the basis of their multilocus genotypes using *Structure* (v. 2.3.4: Pritchard, Stephens, & Donnelly, 2000) with the correlated allele frequencies model (Falush, Stephens, & Pritchard, 2003). For each predefined number (*K* = 1 to 5) of clusters, *Structure* was run with 1 million MCMC iterations following 1 million burnins. The distribution of ln prob(data|K) was evaluated for assessing the most likely number K. Individual *Q*‐values (i.e., the estimated membership coefficients for each individual) were plotted graphically with *Distruct* (Rosenberg, 2004). *Geneclass2* (v.2.0.g: Piry et al., 2004) was used to assign individuals to the aforementioned two reference samples, employing the Bayesian method of Rannala and Mountain (1997).

We used individual cluster memberships, as assigned by *Geneclass2*, and tested for change over time and space in the proportion of eggs and 0‐group juvenile cod that were assigned to the fjord and North Sea reference samples. Under the hypothesis of selective change in genotypic proportions, we expect a decline in proportions of individuals that were assigned to the North Sea population and a corresponding increase in the proportion assigning to the fjord population for samples taken inside the fjords. Such selective shifts, if they exist, must take place largely after the release of eggs to the environment, which occurred around our first sampling date, and before late autumn when the last samples were taken, as these dates span the period with high levels of natural mortality. For Topdalsfjord, one date of eggs (March 5; six sampling sites: Figure 2b) and two temporal replicates of juveniles (June and September) were available for testing (Table 1), resulting in three temporal samples from this fjord. In addition, a sample of adult spawners was available for comparison from the inner part of the Topdalsfjord. For Tvedestrandsfjord, heterogeneity in proportions of the two genotype clusters was tested in five temporal replicate samples for eggs (February 20 to March 24) and two temporal replicates for juveniles (June and October), for a total of seven temporal samples (Table 1).

To test for difference among temporal samples in proportions of individuals assigned to each genetic cluster, we used standard chi‐square heterogeneity tests and regression analyses. We chose logistic regression with *Geneclass2* score as response variable and date of sampling and position of sampling site in the fjord as explanatory variables. The model is logistic because score is a binary variable (1 = individual belong to the North Sea cluster, 0 = individual belong to the fjord cluster) and we used regression because the two explanatory variables are ordinal, and regression is then statistically more powerful than alternative approaches that ignore this information (Agresti, 2013, p. 87). The first explanatory variable was day of sampling, counted as the number of days after the first sampling date, and was taken to represent the time of exposure to the fjord environment. Clearly, this is not exactly so, as eggs may have been spawned at different dates, but these differences should be relatively minor (a few weeks) considering the total time‐span of the study (eight months). The second explanatory variable was sampling position in the fjord (Figure 2: 1 = inner part of fjord, 6 = outer part), which was assumed to represent any of a number of environmental gradients running from the inner to the outer part of the fjords. These gradients could reflect differences in temperature, salinity, oxygen level, prey availability and species composition, parasite prevalence, and so on (cf. Schulze, 2006) that might induce selective mortality on genotypes. The two fjords were analyzed separately, and spawning fish (Topdalsfjord) were not included in the regression analysis, which was based on the following logistic model:

(1) $$s_{i}=\frac{\exp(a+bx_{i}+cy_{i})}{1+\exp(a+bx_{i}+cy_{i})},$$

where the response variable (*s*) is the *Geneclass2* score and explanatory variables (*x* and *y*) are sampling date and station number, respectively, and *i* index individuals. The model parameters (*b* and *c*) were estimated and tested for significance with the *glm* function in the R statistical environment (R Core Team, 2016).

## 3 RESULTS

A total of 409 individuals, representing adults, eggs and juveniles, were genotyped successfully, in the sense that >15 SNPs produced a valid genotype (i.e., <10 SNPs failed). Eggs typically had more missing genotypes than did juveniles and adults, and the number of missing genotypes was greater for eggs with low DNA concentration (Supplementary Information Figure S1). The few eggs that were obtained at the first sampling event, on February 20 in Tvedestrandsfjord, all had very low DNA concentration, presumably reflecting recent spawning (Espeland & Sannæs, 2018). The distribution of egg DNA concentration, and hence age distribution, in Tvedestrandsfjord, was much wider already at the next sampling event a week later (February 27), and by early March tended to be wider than seen in Topdalsfjord at the same date (cf. Supplementary Information Figure S1).

**Figure 3:**
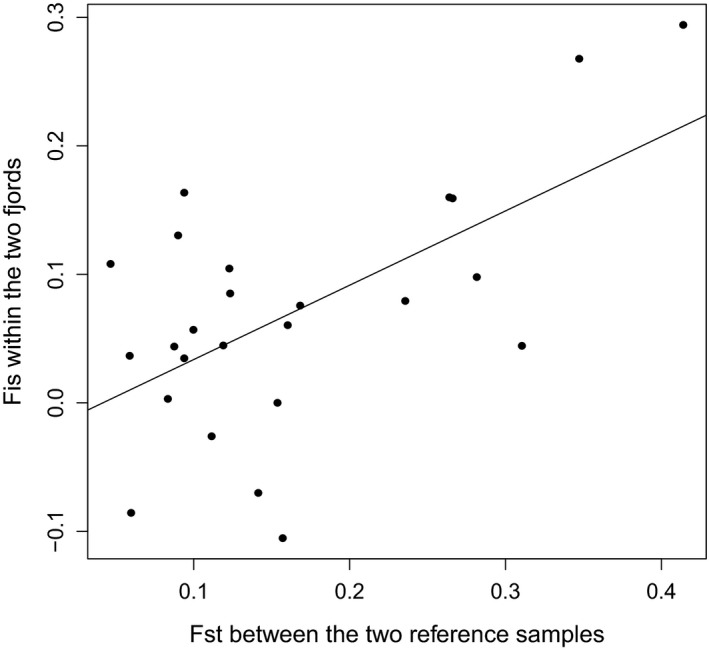
Single‐locus deviations (black dots) from Hardy–Weinberg (*F*_IS_) within fjords (vertical axis) as a function of their level of differentiation (*F*_ST_, horizontal axis) between the two reference samples. Pearson's correlation coefficient *r* = 0.578, *t* = 3.562, *p* = 0.0017. Average *F*_ST_ over all 25 SNPs between the two reference samples was 0.174

Most SNPs displayed a deficiency of heterozygotes in the pooled sample (*n* = 409), with positive *F*_IS_ estimates at 21 out of 25 SNPs (Figure 3). For ten of the SNPs deviation from Hardy–Weinberg (HW) genotype proportions were significant at the 5% level in Tvedestrandsfjord, while three SNPs deviated significantly in Topdalsfjord, two of them in common between fjords. Deficiencies of heterozygotes were also evident from positive average *F*_IS_ estimates in seven out of ten temporal samples from within fjords, two of the ten samples reaching significance at the 5% level (Table 1). The deviations from HW within loci appeared to be linked to the locus’ level of genetic diversity in this geographic region, as single‐locus *F*_IS_ estimates correlated significantly with levels of divergence (*F*_ST_) between the North Sea and fjord reference samples (*r* = 0.578, *p* = 0.0017: Figure 3). The average *F*_ST_ over the 25 SNPs was 0.174 between the fjord and North Sea reference samples and ranged among SNPs from 0.059 to 0.414.

### 3.1 Number of clusters

**Figure 4:**
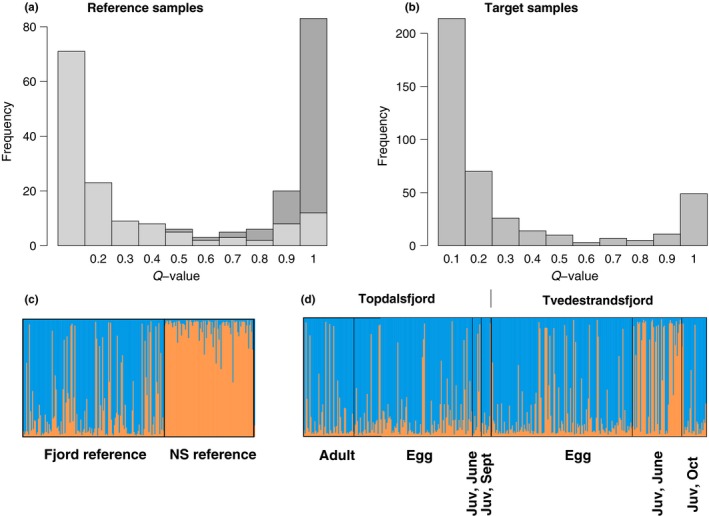
Classification of individual cod into two genetic clusters. Figure panels depict estimated probabilities (*Q*‐values) of individual cod to belong to the North Sea cluster, calculated from 25 SNP genotypes with the *Structure* software. Top panels: (a) frequency histograms for reference samples (light and dark gray for fjord and North Sea reference samples, respectively), and (b) for target samples from the two fjords. (c,d) same data as in a and b, respectively, depicted as individual barplots (orange bars: North Sea cluster; blue bars: fjord cluster), with sample and life stages indicated

Results from *Structure* software were consistent with the existence of two genetic clusters or populations of cod in the samples, with a maximum Ln Prob(data|*K*) for *K* = 2 (Table 2). Estimated membership to either of the *K* = 2 clusters displayed a clear dichotomy with most individuals having either a high (*Q* > 0.8) or a low (*Q* < 0.2) probability of membership to each cluster (Figure 4). Comparison of the Topdalsfjord and Tvedestrandsfjord samples with the two reference samples revealed that the larger of the two clusters coincided with the fjord type (cf. Figure 4c,d) and the smaller cluster with the North Sea type.

**Table 2:** Estimation of number of populations in the combined samples from Topdalsfjord and Tvedestrandsfjord

*K*	Ln Prob(data|*K*)	Prob(*K*|data)
1	−11,409.0	0
2	−10,847.5	1
3	−10,960.8	0
4	−11,150.3	0
5	−11,649.2	0

Note

Numbers depict the log probability of data given various numbers (*K*) of hypothetical clusters or populations, Ln Prob(data|*K*), as reported by *Structure*, and the corresponding estimate of the posterior probabilities of *K*, Prob(*K*|data).

### 3.2 Change in cluster proportions

**Figure 5:**
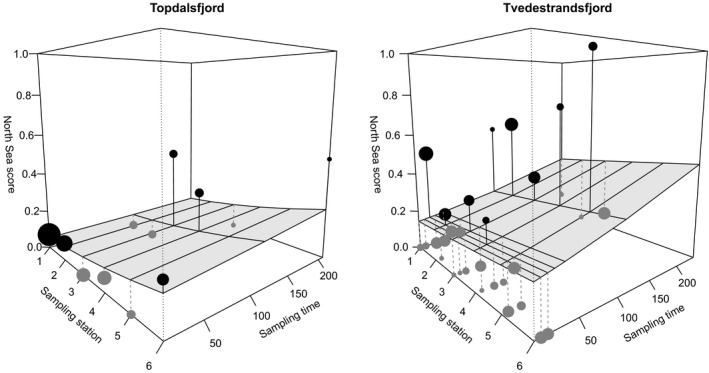
Effects of time (number of days after first sample date) and position in fjord (sample station number) on proportion of individual eggs and juveniles that were scored (*Geneclass2*) to the North Sea type (vertical axes). The shaded plane represents the effects predicted by the model (Equation (1), dots represent data for single samples scaled in proportion to sample size, and other graphical elements are visual aids. Parameter estimates and test statistics are given in Table 3

The test of constant proportions of the two genotype clusters in temporal samples from Topdalsfjord included adults, eggs, early (June) and late (October) juveniles and revealed no difference among life stages (contingency chi‐square test, *p* = 0.511: Table 1). Cod of the putative North Sea type was present in all samples in low proportions, with the highest proportion (two out of seven sampled individuals, or 29%) in the early juvenile sample. The logistic model (Equation (1)) revealed a non‐significant (*p* = 0.148) trend with increasing proportion of the North Sea type toward the outer part of the fjord (higher station number) but little or no change with time (*p* = 0.614; Table 3; Figure 5 left). In Tvedestrandsfjord, which included five replicate egg samples but no adults, there was a highly significant heterogeneity among temporal samples in proportions of the two types (*p* < 0.0001: Table 1). In this locality, heterogeneity was observed both among egg samples (*χ* ^2^ *_df_*_=4_ = 16.14, *p* = 0.0028), between the two juvenile samples (*χ* ^2^ *_df_*_=1_ = 17.593, *p* < 0.0001), with a higher number of North Sea types in the early (June) than in the late (September) juvenile sample (cf. Table 1), and between egg and juvenile samples pooled (*χ* ^2^ *_df_*_=1_ = 30.253, *p* < 0.0001), with a higher proportion of the North Sea type among juveniles than among eggs (42 of 75 = 56% vs. 15 of 143 = 10%). These differences among temporal samples resulted in a statistically significant (*p* = 0.014) increase in North Sea proportions with sampling date in the logistic regression model for this fjord (Table 3; Figure 5 right) but without any clear trend in the spatial dimension (*p* = 0.587). Inspection of the distribution of individual *Structure Q*‐values (Figure 4d) indicated that the observed temporal trend in Tvedestrandsfjord to a large extent reflected an elevated proportion of juveniles of the North Sea type in the June sample; a component that was not seen in the later, October sample.

**Table 3:** The importance of location (station number) and time (date of sampling) on the proportion of individuals assigned to the North Sea reference sample (*Geneclass2* assignments)

Explanatory variable	Topdalsfjord			Tvedestrandsfjord		
	Estimate	*SE*	*p*	Estimate	*SE*	*p*
Station number	0.260	0.180	0.148	0.094	0.133	0.480
Sampling date	0.002	0.005	0.614	0.005	0.002	0.014*

Note

Numbers given are the estimated parameters of the logistic regression model (Equation (1)) for each fjord, with standard errors (*SE*) and *t* tests for significance (*p*: asterisk indicates significance at the 5% level).

## 4 DISCUSSION

Strong selection acting on standing genetic variation could in principle lead to different clusters of genotypes, predominating in different environments, that could be mistaken for genetically differentiated biological populations (cf. Figure 1). If selective survival of members from a common gene pool was responsible for generating genetic clusters of Atlantic cod in Skagerrak coastal waters, the shift in genotypic composition would be expected to take place during a period of strong natural mortality. Given the very high mortality characterizing early life stages in this broadcast spawner, we expected genetic shifts to occur sometime during our first (egg stage) and last (bottom‐settled juvenile fish) sampling times.

In Topdalsfjord, we found no evidence for the predicted genetic changes and members of both clusters were presented in apparently constant proportions during all life stages, including the adult spawners that presumably gave rise to the present offspring cohort. Moreover, the fjord type was the by far most numerous type at all sample times. We therefore reject the hypothesis of selective mortality as an explanation for the observed genetic clusters in this fjord. The situation was more complicated in Tvedestrandsfjord where proportions of the two clusters varied significantly over time, although not in a consistent direction. While temporal samples also in this fjord were dominated by the fjord genetic cluster, episodes of increased presence of individuals of the North Sea cluster occurred both at the egg (in early March) and early juvenile (June) stages. Presumably, these episodes reflected events of inflow of eggs or larvae of North Sea origin into the Tvedestrandsfjord or movement of early juvenile fish. The subsequent decline of North Sea members in later (October) juvenile samples may be suggestive of selective removal of North Sea genotypes in the fjord environment, but cannot explain the dominance of the fjord type already manifested in the earliest, recently spawned egg samples. This latter observation verifies that the two genetic clusters in Tvedestrandsfjord were, as in Topdalsfjord, established already prior to the onset of high natural mortality and potential for strong selection.

If the two genotype clusters are not the result of strong selective survival in different environments of members of the same gene pool they must instead be manifestations of two genetically differentiated lineages or populations, possibly representing different ecotypes with partially overlapping ranges in Skagerrak (Knutsen et al., 2018). This interpretation is consistent with the finding (Figure 3) of a strong correlation among loci in deficiency of heterozygotes and level of genetic divergence, indicating a Wahlund effect (i.e., population mixture) within fjords.

Of the two putative ecotypes, the North Sea type is the only one thus far observed in the North Sea proper (cf. Figure 4c, NS reference sample) and its presence also within fjords may represent drift of pelagic eggs or larvae from the North Sea cod population to the Skagerrak coast (Knutsen et al., 2004; Stenseth et al., 2006). Local spawning of this type on the coast cannot be excluded, however, and nearly 10% (5 out of 52: Table 1) of the adult and presumably mature cod in Topdalsfjord were of this type. We do not know if these individuals actually spawned inside the fjord or represent strayers from other areas, but local spawning of this type could explain why we found apparently very young egg also of the “North Sea” type within fjords (cf. Supplementary Information Figure S1). The drift time from North Sea spawning grounds into the (inner) Skagerrak has been estimated to at least 10 days (Munk et al., 1995).

Since the fjord genetic cluster dominates the inner fjord samples it likely represents a unique lineage of cod. There is evidence that this lineage may be related to the western Baltic cod stock (Barth et al., 2017). Whatever its origin, this type must be largely reproductively isolated from North Sea cod in order to maintain its genetic characteristics where the two types coexist. Apart from the putative indications for selective removal of North Sea cod from within Tvedestrandsfjord, the circumstances allowing co‐occurrence of two types of cod in coastal Skagerrak remain unknown. Similar phenomena of coexisting types have been described for coastal and migratory cod along northern Norway (Johansen et al., 2018; Kirubakaran et al., 2016; Sarvas & Fevolden, 2005; Westgaard & Fevolden, 2007), Iceland (Halldórsdóttir & Árnason, 2015), Greenland (Therkildsen et al., 2013), and Canada (Berg et al., 2017), and thus appear to be common for this species. Phenotypically cryptic, coexisting lineages or ecotypes may be common also in other species but may be under‐reported because their detection requires either highly informative markers or extensive sampling to detect the often weak statistical signals of heterozygote deficiency and admixture linkage disequilibrium (Jorde, Andersson, Ryman, & Laikre, 2018).

A number of studies have explored population genetic differentiation patterns between panels of putative neutral and selected loci and found largely consistent, yet more pronounced differentiation and/or differentiation at finer geographic scales for selected loci (Bekkevold et al., 2015; Larson et al., 2014; Milano et al., 2014). This consistency may be interpreted in support of the notion that selected markers loci represent a valid, and highly informative, tool for population studies in species with low levels of neutral structure. On the other hand, there is little evidence that gene loci generally follow a clear dichotomy into purely neutral and selected classes, and different statistical tools used for discriminating among such locus classes often yield conflicting results (Lotterhos & Whitlock, 2014; Narum & Hess, 2011). The present study does not rely on comparisons of spatial differentiation patterns among putative distinct classes of loci as a means of assessing their reliability as population markers. Instead, our aim was to test the hypothesis that observed differentiation in a high‐graded SNP marker panel might be attributed to recurrent, strong selection.

Despite the high potential for selective shifts of high‐graded SNPs in a species as fecund as the Atlantic cod, we reject this hypothesis. This does not imply that selection on these SNPs or on their linked genomic background is not occurring, but the magnitude of selective mortality during a single season is clearly too small to be detected in the present experimental setting, and also too small to affect the statistical assignment of individuals to population of origin. Hence, this selected SNP panel may be considered valid and highly useful markers for certain population studies, including detection of population subdivisions and assignment of individuals to population of origin. By implication, high‐graded panels should be useful for addressing similar questions also in other areas and for other species, the great majority of which have lower fecundity than the cod and less potential for rapid selective shifts. Of course, due considerations need to be made to the scientific question at hand when employing such a panel.

## CONFLICTS OF INTEREST

The authors declare no conflict of interests.

## AUTHOR CONTRIBUTIONS

This work represents part of AES's Master thesis. The study was designed by HK, AES, PEJ, SEH and MS. AES did the field work, DNA extraction, initial data analyses and wrote the first draft. MS designed the SNP panel. SEH assisted in egg sampling and beach seine hauls. PEJ did the statistical analyses and wrote the present version with assistance from all authors.

## DATA ACCESSIBILITY

SNP genotypes and metadata are available at Dryad https://doi.org/10.5061/dryad.k718h66.
