# Supplementary material for: Can we rely on selected genetic markers for population identification? Evidence from coastal Atlantic cod
Source: Ecol Evol. 2018 Dec 1;8(24):12547–58. doi: 10.1002/ece3.4648 (PMC6308871; doi:10.1002/ece3.4648)
Supplement: Supplementary file 1 [file ECE3-8-12547-s001.doc]

**Supplementary Information**

*Hypothetical selection scenario*

A simple computer simulation was performed to illustrate a hypothetical scenario with strong selection and subsequent genetic analyses. The breeding population was simulated with N=1000 diploid individuals carrying 100 loci with two alleles each. Random drift was simulated in the population over 100 generations in order to randomize genotypes among loci and individuals, using in-house software. The hypothetical scenario was that juveniles from the breeding populations are transported, e.g., by wind or ocean currents, into two nursery areas with differnet environmental conditions and that are subsequently sampled for genetic analyses. The transport and sampling events were simulated by random sampling of 50 individuals from the breeding population to represent nursery area 1 (Fig. 1), i.e. the unselected area. Selection was assumed to operate in nursery area 2 by favouring individuals that were homozygote for allele "1" at each of the 3 first loci (orange dots) and 50 individuals were sampled from the subset of the breeding population that satisfied this condition. The two samples were subsequently analyzed with the *Structure* software and plotted with *Distruct.*


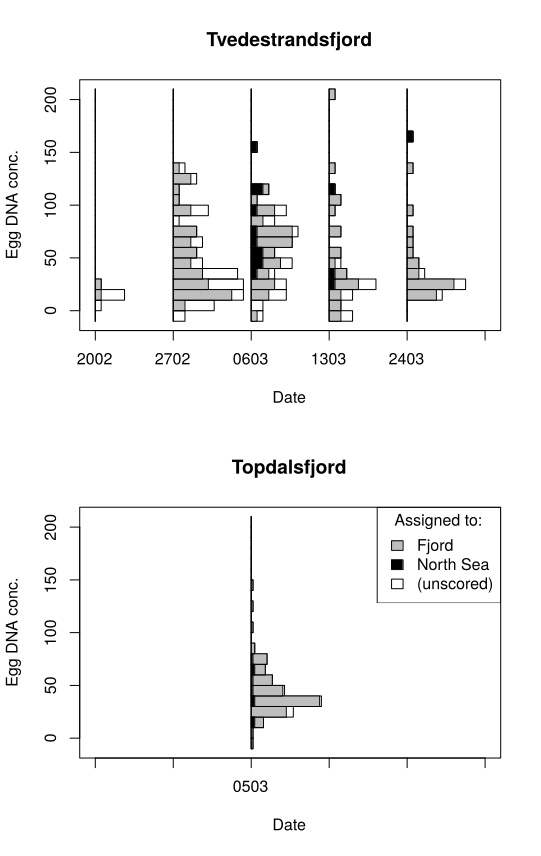


**Figure S1.** Distribution of egg DNA concentration (ug/ml) at different sampling dates (DDMM) in Tvedestrandsfjord (top) and Topdalsfjord (bottom). Histogram bars are shaded according to Geneclass2 assignment to the fjord (gray) and North Sea (black) types, or left white for eggs that were unsuccessfully genotyped (i.e., with 10 or more missing genotypes) or unscored (score percent <80%).
